# Bystin (BYSL) as a possible marker of severe hypoxic-ischemic changes in neuropathological examination of forensic cases

**DOI:** 10.1007/s12024-017-9942-x

**Published:** 2018-01-18

**Authors:** Mieszko Olczak, Dominik Chutorański, Magdalena Kwiatkowska, Dorota Samojłowicz, Sylwia Tarka, Teresa Wierzba-Bobrowicz

**Affiliations:** 1 Department of Forensic Medicine, Medical University of Warsaw, 1 Oczki St., 02-007 Warsaw, Poland; 2 Departament of Neuropathology, Institute of Psychiatry and Neurology, 9 Sobieskiego St., 02-957 Warsaw, Poland

**Keywords:** Bystin, Brain ischemia, Hypoxia-ischemia, Marker

## Abstract

Bystin (BYSL) is a 306-amino acid protein encoded in humans by the BYSL gene located on the 6p21.1 chromosome. It is conserved across a wide range of eukaryotes. BYSL was reported to be a sensitive marker for the reactive astrocytes induced by ischemia/reperfusion and chemical hypoxia in vitro and is considered to be one of the common characteristics of astrogliosis. In our study we examined whether BYSL could be used as a marker for hypoxic-ischemic changes in forensic cases. Groups suspected of acute hypoxic-ischemic changes presented strong BYSL expression in the cytoplasm of neocortical neurons especially in layers 3–5, that seemed to be short-lasting. In the hypoxic-ischemic-reperfusion group we did not find BYSL expression. BYSL expression in the cytoplasm of cortical neurons was minimal in the control group (cardiac arrest). BYSL seems to be a promising early marker of severe hypoxic-ischemic changes in neuropathological examination of forensic cases and certainly requires further studies.

## Introduction

Acute hypoxic-ischemic brain injury results in several biochemical changes, which occur shortly (60–180 s) after its onset. These include ATP depletion, anoxic depolarization and multidirectional impairment of neurolemmal function causing extracellular sodium, chloride and calcium decrease and potassium leak into the extracellular space [1, 2]. Calcium influx into the neuron results in the activation of calcium-dependent pathways (eg. calpain system activation), leading to the onset of apoptosis [3]. Local release of lactic acid and hydrogen ions turns extracellular space acidic within 20 s of the hypoxic/ischemic insult [4]. Glutamate release results in excitotoxic enzyme activation, free radical formation and nitric oxide toxicity leading to neuronal breakdown [5–7].

Those biochemical changes are accompanied by mitochondrial damage [8], abnormal Golgi complexes formation [9], depressed protein synthesis [10] and the induction of heat shock protein production [11]. Neuronal cell death is a result of three coexisting mechanisms: necrotic cell death, apoptosis, and free-radical induced damage with autophagocytosis [12, 13]. Cell death does not occur if the reperfusion is established within the “revival period” [14].

Modern and specific markers of acute hypoxia in human include decreased calbindin-D28k expression in Purkinje-cells [15].

Neurological and neurobehavioral consequences of hypoxic-ischemic brain injury in survivors include: seizures, movement disorders eg. post-hypoxic parkinsonism, delayed post-hypoxic leukoencephalopathy, disturbances of sensorimotor function, cognitive, emotional and behavioral impairments [16, 17].

Bystin (BYSL) is a 306-amino acid protein encoded in humans by the BYSL gene located on the 6p21.1 chromosome also conserved across a wide range of eukaryotes, including mammals [18]. BYSL was originally identified as a cytoplasmic protein forming a complex with thophinin and tastin in human trophoblastic embryonal carcinoma HT-H cells [19, 20]. These proteins are expressed at the uteroplacental interface or at implantation sites at early stages of pregnancy and disappear from the placenta after the 10th week of pregnancy [21]. Analysis of bystin in human cancer cells and mouse embryos indicates that bystin functions in the biogenesis of the 40S ribosome and in cell growth [22, 23]. Animal studies reported presence and up-regulation of BYSL expression levels in astrocytes following brain injury what was promoted by proinflammatory mediators (lipopolisaccharide, interleukin-1β) and nerve growth factor [24]. Compared with GFAP, BYSL was reported to be more sensitive marker for the reactive astrocytes induced by ischemia/reperfusion and chemical hypoxia in vitro and was considered to be one of the common characteristics of astrogliosis [25]. In our study we wanted to examine if BYSL might be a useful marker for hypoxic-ischemic changes in forensic cases.

## Materials and methods

The study was carried out using 9 cases suspected of acute and fatal hypoxic-ischemic changes of the CNS (hanging, carbon monoxide fatal poisoning (HbCO > 50%), fatal external/internal bleeding) - HI group; 7 cases suspected of acute hypoxic-ischemic changes followed by reperfusion and a few (2–5, average of 3.43) days of hospitalization prior to death (hanging, drowning) - HIR group; 8 cases of sudden death - cardiac arrest - sudden and instantaneous death with no cardiopulmonary resuscitation, used as a control group - C group; one case of brain ischemic stroke with proven duration of at least 48 h studied by forensic pathologists at the Department of Forensic Medicine, Medical University of Warsaw.

Brain specimens (frontal lobes) were collected during forensic autopsies and fixed in buffered formalin, then embedded in paraffin. Sections were stained histologically (hematoxylin-eosin) and immunohistochemically with: anti bystin (BYSL) (Merc-Millipore, 1:200), anti-glial fibrillary acidic protein (GFAP) (Serotec, 1:700) and anti-human macrosialin (CD 68) (Dako, 1:75) according to the IHC-P protocols supplied by the manufacturers.

Microphotographs of brain sections were taken with an Olympus BX41 microscope and the Olympus DP25 digital camera. Microphotographs were taken with the same light level for all cases.

To assess the BYSL expression, sections showing BYSL reaction in the frontal cortex were analyzed. Microphotographs of two adjacent areas were taken with magnification of 200 (area of 141,808.92 μm2) for each single case, then converted to a 16-bit grayscale. Percent of positive antibody reaction was counted as a stained area fraction in the analyzed region, with ImageJ 1.41o software and used for statistical analysis. Area fraction was automatically counted with the “Threshold” function of the ImageJ program, which marks all the pixels of a selected grey value and counts all the groups of marked pixels (e.g. positive BYSL reaction) within the selected area. Two adjacent areas for each case were analyzed. Values were averaged for each case and used for statistical analysis [26].

The STATISTICA software package for Windows (Stat-Soft, Tulsa, OK, USA) was used to analyze all data. Probability (P) levels of less than 0.05 were considered significant.

## Results

**Fig. 1:**
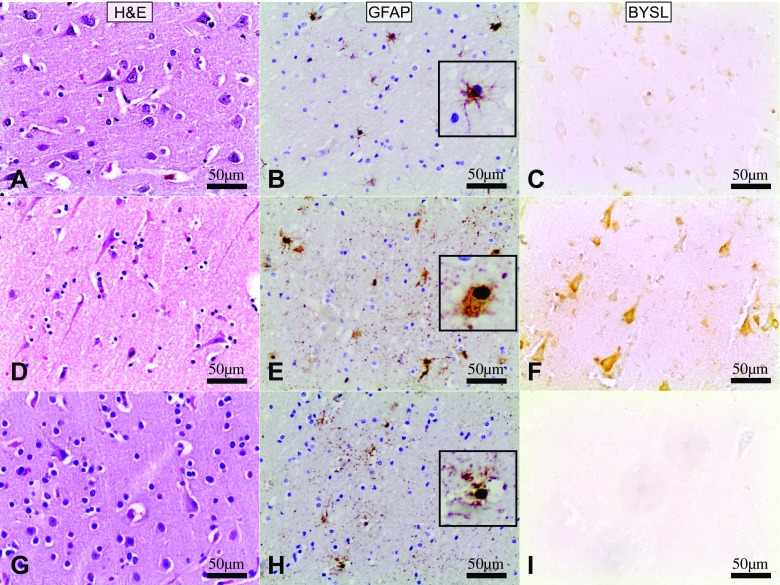
Frontal lobes, magnification × 200. H&E and anti-BYSL staining in the 3rd neuronal layer of the neocortex. GFAP marks white matter. **a** – control group (sudden death), slightly pronounced brain edema; **b** - control group, normal view of astroglia; **c** – control group (sudden death), white matter, minimal BYSL expression in neuron cytoplasm; **d** – HI group - brain edema, ischemic neurons and perineuronal satellitosis; **e** – HI group, astroglia proliferation and clasmatodendrosis; **f** – HI group, white matter, statistically significant (comparing to control group) increased BYSL expression in cytoplasm of ischemic neurons; **g** – HIR group with brain edema, ischemic neurons and severe perineuronal satellitosis; **h** – HIR group, slightly more pronounced astroglia proliferation and clasmatodendrosis; **i** – HIR group with no BYSL expression. Inserts **b**, **e**, **h** shows astrocytes in higher magnification

H&E staining revealed some neuropathological findings in each experimental (HI, HIR) and control (C) groups. Control group presented features of slight to moderate brain edema. The astroglia morphology in GFAP staining appeared normal. BYSL expression in cytoplasm of neurons in cortex was minimal (Fig. 1a–c).

Study groups HI and HIR displayed brain edema and ischemic neurons with perineuronal satellitosis, which was found to be severe in the HIR group in H&E staining (Fig. 1d, g). Both experimental groups presented astroglia proliferation and clasmatodendrosis. Both were slightly more pronounced in HIR group (Fig. 1e, h).

The HI group presented strong BYSL expression in the cytoplasm of neocortical neurons especially in layers 3–5 (Fig. 1f). BYSL expression seemed to be most prominent in ischemic cases (e.g. stab wound with fatal bleeding) and moderate in hypoxic ones (e.g. CO poisoning) (data not shown). In the HIR group we did not find BYSL expression at all (Fig. 1i).

The brains of the control group, and experimental (HI and HIR) groups were quantitatively assessed for BYSL expression. Results presented in Table 1 show that acute hypoxia-ischemia caused statistically significant increase in BYSL expression as compared to the control group and HIR group where no BYSL expression was observed. Mann-Whitney U-test revealed that the amount of BYSL was significantly higher in the IH group than in the control group (*p* < 0.01) and HIR group (*p* < 0.01). The latter (HIR) was also lower than the control group (*p* < 0.01).

**Table 1:** Effect of hypoxia-ischemia on BYSL expression in the 3rd layer of the frontal cortex

Group	AV-AF [%]	±SEM	p/C	p/HIR
C	0,005	0,002	x	<0.01
HI	8704	1485	<0.01	<0.01
HIR	0,000	0,000	<0.01	x

C - control group; HI - acute hypoxia-ischemia group; HIR - acute hypoxia-ischemia with survival/reperfusion group; AV-AF - average stained area fraction (in %) of positive BYSL reaction in the frontal cortex; (sample microphotographs shown on Fig. 1, pictures C, F, I); p/C - significance comparing to control group; p/HIR - significance comparing to HIR group

**Fig. 2:**
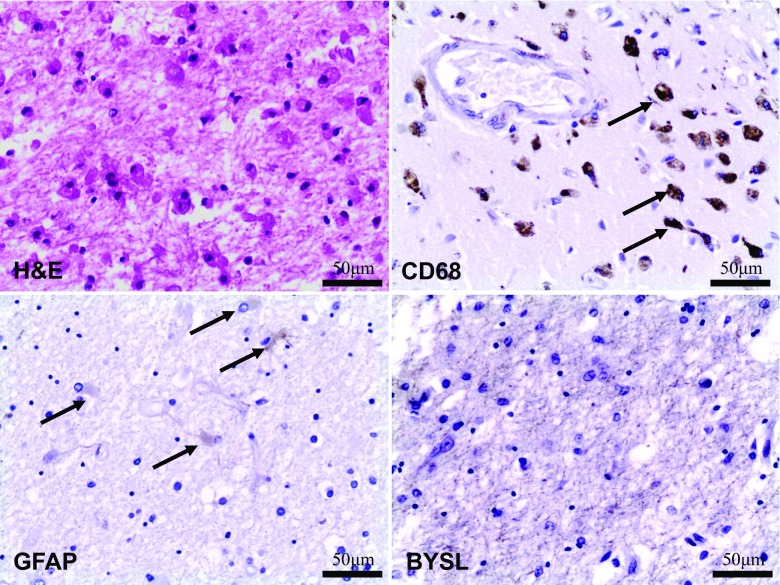
Case of brain ischemic stroke with duration of over 48 h; magnification ×200. H&E - macrophages infiltration; CD68 - macrophages/phagolytic microglia infiltration (arrows) in the vicinity of blood vessel; GFAP - astrocytes (arrows) in the area of brain ischemic stroke, poor GFAP expression; BYSL - no BYSL expression in the area of brain ischemic stroke

As we found no BYSL expression in the hypoxic-ischemic changes followed by reperfusion (HIR) group, we performed additional staining to confirm that finding. We examined a case of brain ischemic stroke with proven duration of over 48 h. No BYSL expression was found in this case, similar to the HIR group. Sample microphotographs are shown on Fig. 2.

## Discussion

Hypoxic-ischemic injury includes number of neuropathological changes seen in the light microscope examination. Within 1 h of the insult microvacuolization with normal or shrunken nucleus, cell shrinkage, normal or slightly basophilic cytoplasm, and perineuronal spaces around some neurons may be observed. These changes cannot be distinguished from postmortem autolysis [27]. For this reason we proposed BYSL as a new marker of early ongoing neuronal changes after hypoxic-ischemic episodes.

Typical neuropathological hypoxic-ischemic changes include: ischemic cell change - seen in the first 6 h of survival, with further neuronal nucleus and cytoplasm shrinkage and pink cytoplasm staining; homogenizing cell change typically seen around 24 h after survival; and a final stage termed “ghost cell” with reduced or absent cytoplasm around a dark and shrunken nucleus and eventual cell loss [27]. In our HI group ischemic neurons were present despite the fact that death occurred immediately after the ischemic episode. This further emphasizes a need for a validated early marker of hypoxic-ischemic changes.

In our study we documented that acute and fatal hypoxic-ischemic brain injury results in increased BYSL expression in cortical neurons, especially in layers 3–5. Similar findings were documented previously *in vitro* [25] and in animal studies [24]. BYSL expression seems to be more prominent in ischemic episodes (e.g. fatal bleeding) and moderate in hypoxic ones (e.g. CO poisoning), but it is possible that the length of the episode plays an important role here. Minimal BYSL expression in control group shows, that ischemic brain injury in cardiac arrest may also be distinguished and recognized with this technique. Previous studies describe minimal BYSL expression in astrocytes of cerebral cortex in a physiologically “normal” CNS [24]. Similarly, previous studies showed that BYSL expression is a more sensitive marker for reactive astrocytes and can be considered one of the common characteristics of astrogliosis [25]. Here we documented BYSL as a potential marker of hypoxic-ischemic brain injury with a short survival time after the episode, which could be useful for future forensic examinations. In spite of GFAP expression not revealing differences between hypoxic-ischemic and hypoxic-ischemic-reperfusion groups, BYSL expression was increased and detectable (within ≤48 h) after the hypoxic-ischemic episode, which could be potentially useful for dating of hypoxic-ischemic episodes. Further studies including animal ones are needed to establish the precise dynamics of BYSL expression. Previous studies prompted a hypothesis that cell death results in decrease of BYSL expression [25]. This hypothesis, however, requires further studies. In *in vitro* models 0.5 and 3 h ischemia induced significant BYSL astrocyte expression while no differences between the control group and 24 h ischemia and 24 h ischemia/reperfusion were observed. Similarly mild chemical hypoxia induced significant BYSL expression *in vitro*. These increases were not accompanied by increased GFAP expression [25]. In experimental animal models of brain injury elevated BYSL level was observed more than 5 weeks after the injury [24] and it was suggested that BYSL may be involved in glia activation and differentiation as its increased expression was observed *in vitro* during astrocyte IL-β transformation [24]. Studies *in vitro* showed increased BYSL expression was observed positively correlated with IL-1β, IFN-γ, NGF action [24].

## Conlusion

BYSL seems to have a great potential, as an early marker of severe hypoxic-ischemic changes in neuropathological examination of forensic cases and certainly requires further studies.

## Key points

1. Hypoxia-ischemia leads to enhanced, short-lasting induction of bystin expression in cytoplasm of neocortical neurons.
2. No bystin expression was observed in the hypoxic-ischemic-reperfusion group.
3. Cardiac arrest leads to a minimal bystin expression in cortical neurons.
4. Bystin may serve as an early marker of severe hypoxic-ischemic changes in neuropathological examination of forensic cases.
